# Mendelian Randomization Study of B-Type Natriuretic Peptide and Type 2 Diabetes: Evidence of Causal Association from Population Studies

**DOI:** 10.1371/journal.pmed.1001112

**Published:** 2011-10-25

**Authors:** Roman Pfister, Stephen Sharp, Robert Luben, Paul Welsh, Inês Barroso, Veikko Salomaa, Aline Meirhaeghe, Kay-Tee Khaw, Naveed Sattar, Claudia Langenberg, Nicholas J. Wareham

**Affiliations:** 1 Medical Research Council Epidemiology Unit, Institute of Metabolic Science, University of Cambridge, Cambridge, United Kingdom; 2 Department III of Internal Medicine, Heart Centre of the University of Cologne, Cologne, Germany; 3 Department of Public Health and Primary Care, Institute of Public Health, University of Cambridge, Cambridge, United Kingdom; 4 Division of Cardiovascular and Medical Sciences, University of Glasgow, Glasgow, United Kingdom; 5 Metabolic Disease Group, Wellcome Trust Sanger Institute, Wellcome Trust Genome Campus, Hinxton, United Kingdom; 6 University of Cambridge Metabolic Research Laboratories, Institute of Metabolic Science, Addenbrooke's Hospital, Cambridge, United Kingdom; 7 Department of Chronic Disease Prevention, National Institute for Health and Welfare, Helsinki, Finland; 8 INSERM U744, Institut Pasteur de Lille, Université Lille Nord de France, UDSL, Lille, France; Peninsula Medical School, United Kingdom

## Abstract

**Background:**

Genetic and epidemiological evidence suggests an inverse association between B-type natriuretic peptide (BNP) levels in blood and risk of type 2 diabetes (T2D), but the prospective association of BNP with T2D is uncertain, and it is unclear whether the association is confounded.

**Methods and Findings:**

We analysed the association between levels of the N-terminal fragment of pro-BNP (NT-pro-BNP) in blood and risk of incident T2D in a prospective case-cohort study and genotyped the variant rs198389 within the BNP locus in three T2D case-control studies. We combined our results with existing data in a meta-analysis of 11 case-control studies. Using a Mendelian randomization approach, we compared the observed association between rs198389 and T2D to that expected from the NT-pro-BNP level to T2D association and the NT-pro-BNP difference per C allele of rs198389. In participants of our case-cohort study who were free of T2D and cardiovascular disease at baseline, we observed a 21% (95% CI 3%–36%) decreased risk of incident T2D per one standard deviation (SD) higher log-transformed NT-pro-BNP levels in analysis adjusted for age, sex, body mass index, systolic blood pressure, smoking, family history of T2D, history of hypertension, and levels of triglycerides, high-density lipoprotein cholesterol, and low-density lipoprotein cholesterol. The association between rs198389 and T2D observed in case-control studies (odds ratio = 0.94 per C allele, 95% CI 0.91–0.97) was similar to that expected (0.96, 0.93–0.98) based on the pooled estimate for the log-NT-pro-BNP level to T2D association derived from a meta-analysis of our study and published data (hazard ratio = 0.82 per SD, 0.74–0.90) and the difference in NT-pro-BNP levels (0.22 SD, 0.15–0.29) per C allele of rs198389. No significant associations were observed between the rs198389 genotype and potential confounders.

**Conclusions:**

Our results provide evidence for a potential causal role of the BNP system in the aetiology of T2D. Further studies are needed to investigate the mechanisms underlying this association and possibilities for preventive interventions.

*Please see later in the article for the Editors' Summary*

## Abstract 2

Using mendelian randomization, Roman Pfister and colleagues demonstrate a potentially causal link between low levels of B-type natriuretic peptide (BNP), a hormone released by damaged hearts, and the development of type 2 diabetes.

## Editors' Summary

**Background:**

Worldwide, nearly 250 million people have diabetes, and this number is increasing rapidly. Diabetes is characterized by dangerous amounts of sugar (glucose) in the blood. Blood sugar levels are normally controlled by insulin, a hormone that the pancreas releases after meals (digestion of food produces glucose). In people with type 2 diabetes (the most common form of diabetes), blood sugar control fails because the fat and muscle cells that usually respond to insulin by removing sugar from the blood become insulin resistant. Type 2 diabetes can be controlled with diet and exercise, and with drugs that help the pancreas make more insulin or that make cells more sensitive to insulin. The long-term complications of diabetes, which include kidney failure and an increased risk of cardiovascular problems such as heart disease and stroke, reduce the life expectancy of people with diabetes by about 10 years compared to people without diabetes.

**Why Was This Study Done?:**

Because the causes of type 2 diabetes are poorly understood, it is hard to devise ways to prevent the condition. Recently, B-type natriuretic peptide (BNP, a hormone released by damaged hearts) has been implicated in type 2 diabetes development in cross-sectional studies (investigations in which data are collected at a single time point from a population to look for associations between an illness and potential risk factors). Although these studies suggest that high levels of BNP may protect against type 2 diabetes, they cannot prove a causal link between BNP levels and diabetes because the study participants with low BNP levels may share some another unknown factor (a confounding factor) that is the real cause of both diabetes and altered BNP levels. Here, the researchers use an approach called “Mendelian randomization” to examine whether reduced BNP levels contribute to causing type 2 diabetes. It is known that a common genetic variant (rs198389) within the genome region that encodes BNP is associated with a reduced risk of type 2 diabetes. Because gene variants are inherited randomly, they are not subject to confounding. So, by investigating the association between BNP gene variants that alter NT-pro-BNP (a molecule created when BNP is being produced) levels and the development of type 2 diabetes, the researchers can discover whether BNP is causally involved in this chronic condition.

**What Did the Researchers Do and Find?:**

The researchers analyzed the association between blood levels of NT-pro-BNP at baseline in 440 participants of the EPIC-Norfolk study (a prospective population-based study of lifestyle factors and the risk of chronic diseases) who subsequently developed diabetes and in 740 participants who did not develop diabetes. In this prospective case-cohort study, the risk of developing type 2 diabetes was associated with lower NT-pro-BNP levels. They also genotyped (sequenced) rs198389 in the participants of three case-control studies of type 2 diabetes (studies in which potential risk factors for type 2 diabetes were examined in people with type 2 diabetes and matched controls living in the East of England), and combined these results with those of eight similar published case-control studies. Finally, the researchers showed that the association between rs198389 and type 2 diabetes measured in the case-control studies was similar to the expected association calculated from the association between NT-pro-BNP level and type 2 diabetes obtained from the prospective case-cohort study and the association between rs198389 and BNP levels obtained from the EPIC-Norfolk study and other published studies.

**What Do These Findings Mean?:**

The results of this Mendelian randomization study provide evidence for a causal, protective role of the BNP hormone system in the development of type 2 diabetes. That is, these findings suggest that low levels of BNP are partly responsible for the development of type 2 diabetes. Because the participants in all the individual studies included in this analysis were of European descent, these findings may not be generalizable to other ethnicities. Moreover, they provide no explanation of how alterations in the BNP hormone system might affect the development of type 2 diabetes. Nevertheless, the demonstration of a causal link between the BNP hormone system and type 2 diabetes suggests that BNP may be a potential target for interventions designed to prevent type 2 diabetes, particularly since the feasibility of altering BNP levels with drugs has already been proven in patients with cardiovascular disease.

**Additional Information:**

Please access these websites via the online version of this summary at http://dx.doi.org/10.1371/journal.pmed.1001112.

- The International Diabetes Federation provides information about all aspects of diabetes
- The US National Diabetes Information Clearinghouse provides detailed information about diabetes for patients, health-care professionals, and the general public (in English and Spanish)
- The UK National Health Service Choices website also provides information for patients and carers about type 2 diabetes and includes people's stories about diabetes
- MedlinePlus provides links to further resources and advice about diabetes (in English and Spanish)
- Wikipedia has pages on BNP and on Mendelian randomization (note: Wikipedia is a free online encyclopedia that anyone can edit; available in several languages)
- The charity Healthtalkonline has interviews with people about their experiences of diabetes; the charity Diabetes UK has a further selection of stories from people with diabetes

## Introduction

B-type natriuretic peptide (BNP) is a hormone released from the myocardium in response to increased mechanical strain in order to maintain cardiac function by mediating vasodilation, natriuresis, and anti-fibrotic effects [1]. In addition to cardiovascular effects, BNP also has lipolytic activity in human adipose tissue [2]. This—together with the observation that levels of BNP or the inactive fragment of its pro-hormone (N-terminal fragment of pro-BNP [NT-pro-BNP]) are consistently decreased in individuals with obesity, insulin resistance, and type 2 diabetes (T2D) in cross-sectional studies—raises the possibility of a role of BNP in the aetiology of metabolic disease [3],[4].

Additional evidence suggests a specific link between a common genetic variant (rs198389) within the BNP gene locus (*NPPB*) and risk of T2D [5]. Importantly, a variant in high linkage disequilibrium with rs198389 (rs632793 within the adjacent locus *NPPA*, *r* ^2^ = 0.87) was shown to be associated with BNP/NT-pro-BNP levels in a meta-analysis of four cohorts comprising more than 14,000 individuals; the allele associated with a lower T2D risk was associated with higher BNP/NT-pro-BNP levels [6]. This might point to a potential beneficial effect of BNP hormone in the aetiology of T2D.

However, there is also evidence to suggest reverse causality, with BNP/NT-pro-BNP levels being a consequence rather than a cause of T2D. Short-term increase of insulin levels was shown to decrease NT-pro-BNP levels in non-diabetic individuals with ischemic heart disease [7]. Additionally, obesity somehow impairs the BNP/NT-pro-BNP response [8]–[10]. Both mechanisms might lead to an overestimation of the association between BNP/NT-pro-BNP and T2D risk in cross-sectional analysis.

So far, to our knowledge, only one prospective study has examined the association between BNP/NT-pro-BNP levels and incident T2D [11]. However, in this study 5% of participants reported cardiovascular disease at baseline. Individuals with cardiovascular disease show up to 100-fold increased blood levels of BNP/NT-pro-BNP compared to healthy individuals, which might lead to a distorted estimate of the BNP/NT-pro-BNP to T2D association.

The aim of our study was to investigate the causal role of BNP in the aetiology of T2D by using a Mendelian randomization approach. Therefore, we analysed the association between NT-pro-BNP levels and incident T2D in a large prospective case-cohort study excluding individuals with baseline T2D and cardiovascular disease. We then extended existing genetic data by de novo genotyping of the variant rs198389 in three T2D case-control studies and by using unpublished data from the Diabetes Genetics Replication and Meta-Analysis+(DIAGRAM+) consortium, together comprising 15,638 T2D cases and 47,559 controls. Finally, we estimated the expected association between rs198389 and T2D risk based on the NT-pro-BNP to T2D association and the difference in NT-pro-BNP levels associated with each rs198389 C allele, and performed instrumental variable analysis to calculate the unconfounded effect size of NT-pro-BNP levels on T2D risk.

## Methods

### Ethics Statement

The Cambridgeshire and the ADDITION-Ely case-control studies received ethical approval from the Cambridge Local Research Ethics Committee, and participants provided informed consent. The EPIC-Norfolk cohort study was approved by the Norwich Local Research Ethics Committee.

### Study Populations

We used three T2D case-control studies (Cambridgeshire, ADDITION-Ely, and Norfolk Diabetes) and the population-based EPIC-Norfolk cohort for genetic analysis, and the EPIC-Norfolk cohort for blood-based analysis. De novo genotyping of the variant rs198389 was performed in the three case-control studies comprising 7,508 cases and 8,572 controls, and in the total EPIC-Norfolk cohort. Additionally, we used unpublished genetic data from T2D case-control sets of the DIAGRAM+ consortium.

#### Cambridgeshire case-control study

The Cambridgeshire case-control study is a population-based study of T2D cases, aged 45–76 y, and age- and sex-matched controls. Cases were randomly selected from general practitioner diabetes registers in Cambridgeshire, UK, and T2D was defined as onset of diabetes after the age of 30 y and without insulin use in the first year after diagnosis [12]. Controls were recruited at random from the same population sampling frames, and individually matched to cases for age, sex, and general practitioner practice. Diabetes was excluded in controls by medical record search and by a glycated haemoglobin measurement of less than 6%. In the current analyses, we include 506 cases and 512 controls, representing all white Europeans who had DNA available and information on body mass index (BMI).

#### ADDITION-Ely case-control study

Previously undiagnosed prevalent cases of T2D, defined using World Health Organization oral glucose tolerance testing criteria, were identified via a population-based stepwise screening strategy among 40- to 69-y olds participating in the UK Cambridge arm of the ADDITION study. Current analyses include 765 white European men and women who had DNA available and information on BMI [13]. Controls were identified from the Medical Research Council (MRC) Ely study, a population-based cohort of white European men and women aged 35 to 79 y without diagnosed diabetes and from a similar sampling frame as the cases [14]. Based on WHO oral glucose tolerance testing criteria, participants were confirmed as controls (*n* = 1,606) or classified as cases (*n* = 91).

#### Norfolk Diabetes case-control study

The Norfolk Diabetes case-control study is a study of men and women with T2D in Norfolk, UK. All T2D patients identified through general practice diabetes registers in Norfolk and local hospital diabetes clinic and retinal screening programme patient registers were invited to participate; a total of 6,146 white European cases were included in the current analyses, aged 31 to 98 y. Participants with insulin use during the first year of diagnosis, and those with cystic fibrosis, chronic pancreatitis, or long-term steroid use were excluded from the study. The 6,454 controls free of known diabetes at baseline and during follow-up were randomly selected from participants of the EPIC-Norfolk cohort, who are described in more detail below.

#### Case-control dataset of the DIAGRAM+ consortium

We used the unpublished pooled effect estimate of rs198389 on T2D risk from the published dataset of eight case-control studies comprising 8,130 T2D patients and 38,987 controls of European descent reflecting the total stage 1 dataset of the DIAGRAM+ consortium (for details of the eight cohorts [Wellcome Trust Case Control Consortium, Diabetes Genetics Initiative, Finland-US Investigation of NIDDM Genetics, deCODE Genetics, Diabetes Gene Discovery Group, Cooperative Health Research in the Region of Augsburg, Rotterdam Study, and European Special Population Research Network] see electronic supplementary material Table 1 of [15]). All stage 1 samples of DIAGRAM+ had genome-wide association data available and hence allowed in silico analysis. The effective sample size was *n* = 22,044. Association estimates of the eight individual studies were combined by fixed-effects, additive-model meta-analysis using the inverse-variance method.

#### EPIC-Norfolk cohort study

EPIC-Norfolk is a prospective cohort study in which men and women aged 39 to 79 y were recruited from general practices in the Norfolk region, UK. Full details of the population are reported elsewhere [16]. Between 1993 and 1997, 25,639 participants completed a health and lifestyle questionnaire and a health examination; non-fasting blood samples were taken for analysis of laboratory markers. Details of assessment of baseline variables are described elsewhere [17],[18]. DNA from stored baseline blood samples was available for genotyping in 21,121 participants.

We used a case-cohort study for incident T2D nested within the total EPIC-Norfolk cohort to assess the association between NT-pro-BNP levels and incident T2D. All individuals with any evidence of diabetes at baseline were excluded. Prevalent diabetes was identified on the basis of baseline self-report of a history of diabetes, doctor-diagnosed diabetes, diabetes drug use, or evidence of diabetes after baseline with a date of diagnosis earlier than the baseline recruitment date. Ascertainment of incident T2D in the EPIC-Norfolk cohort used multiple sources of evidence including self-report (self-reported history of T2D, doctor-diagnosed T2D, diabetes drug use), linkage to primary care registers, secondary care registers, hospital admissions, and mortality data. To increase the specificity of the case definition, we sought further evidence for all cases with information on incident T2D from two independent sources at a minimum, including individual medical record review and diabetes register searches. Follow-up was censored at the date of diagnosis, 31 December 2007, or the date of death, whichever occurred first. Individuals without stored blood or without information on reported diabetes status were excluded, leaving a total of 661 incident T2D cases.

In our case-cohort design we randomly selected a subcohort of 877 individuals from those with available stored blood. By design, this subcohort also included a random set of 24 individuals who had developed incident T2D during follow-up, i.e., the case-cohort set included 661 incident T2D cases and 853 non-cases. For this analysis we excluded individuals with history of cardiovascular disease defined by self-reported myocardial infarction or stroke (*n* = 88), individuals without NT-pro-BNP measure (*n* = 132), and individuals without information on covariates for multivariable analysis (*n* = 114), leaving 440 incident T2D cases and 740 non-cases.

In addition, we used 6,454 participants randomly selected from the total EPIC-Norfolk cohort without known or incident diabetes and available baseline DNA samples as controls in the Norfolk Diabetes case-control study. Of these, 650 were also non-cases in the subcohort and were used to examine the association between the variant rs198389 and NT-pro-BNP levels.

### Measurement of Serum NT-pro-BNP Levels

NT-pro-BNP was measured on stored baseline serum samples in the T2D case-cohort study of the EPIC-Norfolk cohort (440 cases/740 non-cases) using an electrochemiluminescence immunoassay on the Elecsys 2010 Immunoanalyzer (Roche Diagnostics). The assay has an effective measuring range of 5–35,000 pg/ml. The median within-run coefficient of variation was 4.5% at a concentration of 129 pg/ml and 1.0% at 4,538 pg/ml. The overall coefficients of variation throughout the study were 4.6% and 4.8% at the same concentrations.

### Genotyping

We genotyped the rs198389 genetic variant within the “natriuretic peptide precursor B” locus, reported to be associated with T2D risk in a candidate gene study and replicated in a meta-analysis [5],[19]. For the total EPIC-Norfolk cohort, genotyping was performed by using an iPLEX (Sequenom) platform. For the three case-control studies, genotyping was performed with Custom TaqMan SNP Genotyping Assays (Applied Biosystems). The variant passed quality-control criteria (call rate >95% and duplicate concordance >98% assessed in approximately 1% of each study sample) and was in Hardy-Weinberg equilibrium (*p*-values>0.05) in all cohorts. Allele frequencies were consistent with those reported for the CEU population (US residents with northern and western European ancestry of the Centre d'Etude du Polymorphisme Humain) of the HapMap database (http://hapmap.ncbi.nlm.nih.gov/cgi-perl/gbrowse/hapmap28_B36/).

### Statistical Analyses

#### Association of BNP levels with T2D risk

The prospective association between log-transformed NT-pro-BNP levels and incident T2D was examined in the case-cohort study of the EPIC-Norfolk cohort, baseline characteristics of which are shown in Table S1. We used Prentice-weighted Cox models (with age as underlying time scale) to account for the case-cohort design [20]. We did not observe evidence for a statistical departure from linearity in the NT-pro-BNP level to T2D association when calculating the difference in log likelihood for modelling quartiles of log-transformed NT-pro-BNP levels as a continuous term against categories (*p* = 0.11, data not shown). Hazard ratios (HRs) are presented, representing the effect of a change of one sex-specific standard deviation (SD) in log-transformed NT-pro-BNP levels. We combined our estimate with the results of a study that was published while our work was ongoing [11], applying a fixed-effects meta-analysis model as no evidence for heterogeneity between the two studies was observed. In the original report of the additional study, NT-pro-BNP was cubic-root-transformed for analysis. For our meta-analysis we used estimates that were re-calculated with log-transformation instead.

#### Association of rs198389 with BNP levels and cardio-metabolic traits

The association between the variant rs198389 and log-transformed NT-pro-BNP levels was examined in the EPIC-Norfolk subcohort, excluding participants with incident T2D. As described previously [6], residuals were obtained using sex-specific regression models in which log-transformed NT-pro-BNP concentrations were adjusted for age, BMI, and hypertension. We observed an additive effect for the association between the rs198389 alleles and serum NT-pro-BNP levels, and genetic variant was coded as 0, 1, and 2 on the basis of the number of the serum NT-pro-BNP-increasing alleles (C alleles).

We identified published studies reporting levels of BNP/NT-pro-BNP by genetic variants within the BNP locus by performing a systematic literature search in PubMed 2.0 (US National Library of Medicine) using the search terms “single nucleotide polymorphism” and “natriuretic peptide” (last search conducted 1 December 2010). Review of 60 identified abstracts revealed four studies that reported BNP/NT-pro-BNP levels by BNP genotype. We excluded one study that examined a population of non-European descent [21] and two studies that examined individuals with advanced cardiac disease [5],[22]. One study was a meta-analysis of four cohorts of European descent that reported the association between the genetic variant rs632793 (linkage disequilibrium with rs198389, *r* ^2^ = 0.87) and BNP or NT-pro-BNP levels [6]. We used a random effect model to update this meta-analysis when including our results, as we observed evidence for significant heterogeneity across studies.

One key assumption of Mendelian randomization is that the genetic variants do not show pleiotropic effects, i.e., are not associated with other diabetes risk factors. To test this assumption the association between the variant rs198389 and cardio-metabolic traits was examined in participants of the EPIC-Norfolk cohort study without prevalent T2D (maximum *n* = 19,746).

#### Association between rs198389 and T2D risk

Logistic regression analysis was used to calculate odds ratios (ORs) for the association between rs198389 and T2D, adjusting for age, sex, and BMI unless indicated otherwise. As observed for the association with NT-pro-BNP levels, there was evidence for an additive effect of rs198389 on T2D risk within our three case-control studies, with an OR of 0.91 (95% CI 0.84–0.99) and 0.87 (95% CI 0.78–0.97) for the CT and the CC genotype compared to the TT genotype, and with the lowest *p*-value (*p* = 0.007) for the additive model compared to dominant (*p* = 0.008) and recessive (*p* = 0.10) models. ORs of the three case-control studies (Cambridgeshire, ADDITION-Ely, and Norfolk Diabetes, together comprising 7,508 cases and 8,572 controls) were combined with the pooled estimate of the DIAGRAM+ consortium comprising 8,130 T2D cases and 38,987 controls of European descent [15] and seven additional case-control studies of an existing meta-analysis comprising 7,744 T2D cases and 10,339 controls of European descent [19] by applying a fixed-effects meta-analysis model. The latter meta-analysis was identified in a systematic literature research in PubMed 2.0 using the search terms “single nucleotide polymorphism”, “natriuretic peptide”, and “diabetes mellitus” (last search conducted 1 December 2010). Review of five identified abstracts revealed two meta-analyses on the effect of the variant rs198389 on T2D in cohorts of European descent, one of which was the update [19] of the other one [5]. We excluded five of the 12 case-control studies reported in the meta-analysis [19] because they were part of DIAGRAM+ (Diabetes Genetics Initiative, Finland-US Investigation of NIDDM Genetics, and deCODE Genetics) or because they were earlier versions of our case-control studies with smaller sample sizes (Norfolk Diabetes and Cambridgeshire).

**Figure 1:**
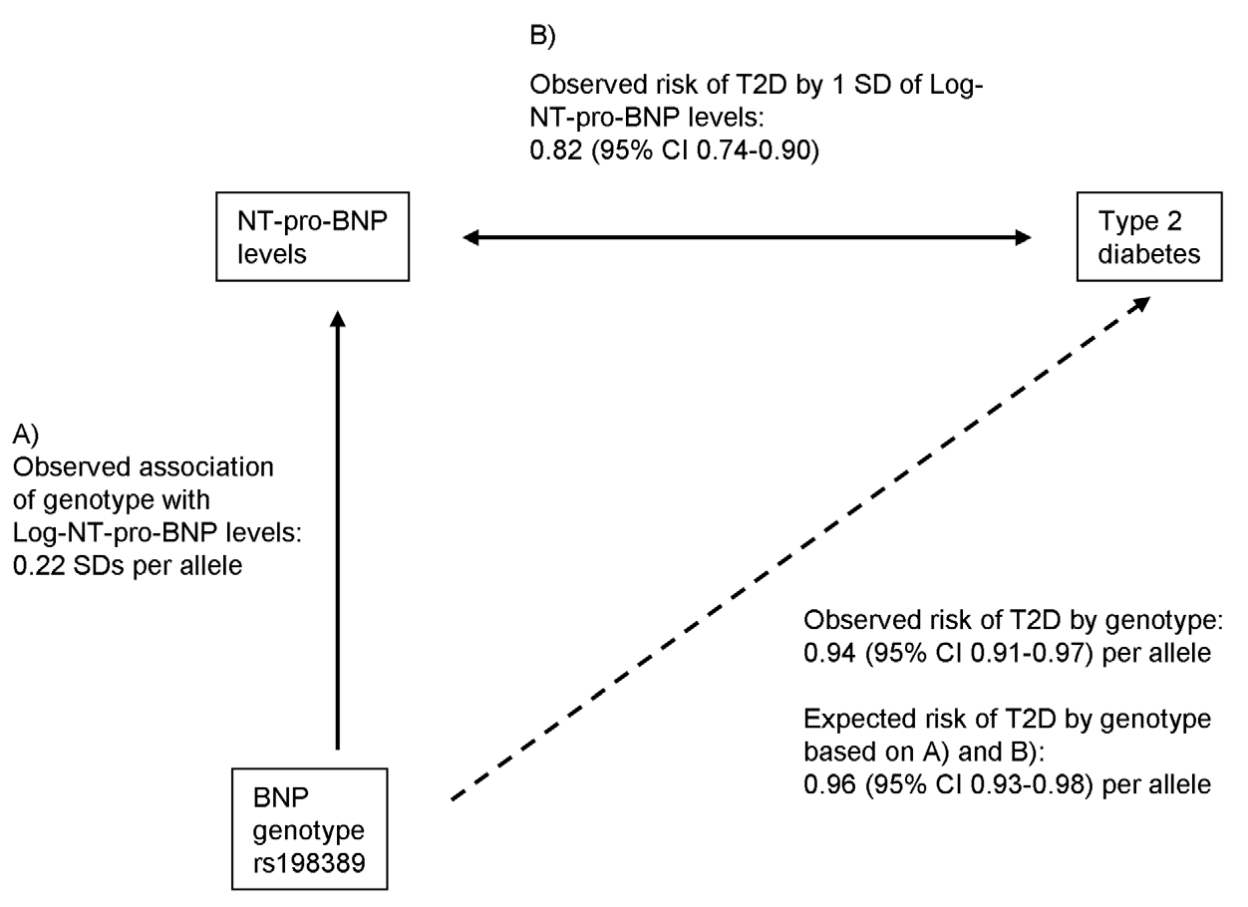
Mendelian randomization approach for the association between BNP and T2D. The observed association between BNP genotype rs198389 and risk of T2D is compared with that expected based on the genotype to peptide level association and the peptide level to T2D association.

The concept of Mendelian randomization is used to test causality of associations between risk factors and outcomes [23]. Genetic variants are randomly allocated during gamete formation, and hence are not subject to environmental influences or reverse causation. Accordingly, genetic variants that are associated with the risk factor can be used as instrumental variables to calculate an estimate of the magnitude of association free of the problems of confounding and reverse causality. We used the estimates of the association of the genetic variant rs198389 with serum NT-pro-BNP levels (see A in Figure 1) and of the association of serum NT-pro-BNP levels with T2D (see B in Figure 1) to calculate an approximate expected effect of the genetic variant on T2D, assuming an aetiological role of BNP levels for T2D:
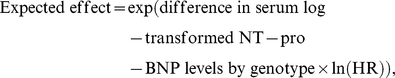
where HR is the risk of incident T2D per SD of log-transformed NT-pro-BNP level. The standard error for the expected effect size was calculated using a Taylor series approximation [24]. Finally, we also performed an instrumental variable analysis using a logistic control function estimator to estimate the unconfounded effect size of log-transformed NT-pro-BNP levels on T2D risk using individuals of the T2D case-cohort study of the EPIC-Norfolk cohort who also had the rs198389 genotype available (*n* = 623 non-cases, *n* = 371 cases). As previously described [25], in this analysis the variation of the potentially causal risk factor NT-pro-BNP that is determined by the instrument (rs198389) is related to the risk of T2D by applying a two-stage analysis. In the first stage, the observational association between genotype and log-transformed NT-pro-BNP was estimated in linear regression; in the second stage the predicted values and residuals from this model were included as covariates in a logistic regression model with T2D as the outcome.

All analyses were performed using STATA version 10.1 (Statacorp).

## Results

### Association of B-Type Natriuretic Peptide Levels with Type 2 Diabetes Risk

NT-pro-BNP levels were inversely associated with risk of T2D in age- and sex-adjusted analysis of the EPIC-Norfolk case-cohort (440 cases/740 controls), with a HR of 0.82 (95% CI 0.69–0.97, *p* = 0.02) for the difference of 1 SD in log-transformed NT-pro-BNP levels. In multivariable analysis adjusting for age (underlying time scale), sex, family history of diabetes, systolic blood pressure, BMI, current cigarette smoking, levels of high-density lipoprotein and low-density lipoprotein cholesterol and triglycerides, and history of hypertension, every increase of one SD in log-transformed NT-pro-BNP levels was associated with a 21% decreased risk of T2D (HR = 0.79, 95% CI 0.64–0.97, *p* = 0.02), with similar results in men (HR = 0.82, 95% CI 0.57–1.18) and women (HR = 0.76, 95% CI 0.60–0.96) and without evidence for a significant sex by NT-pro-BNP interaction (*p* = 0.48).

**Figure 2:**
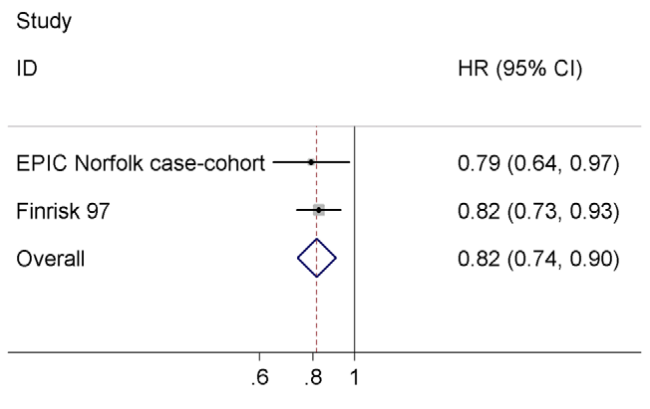
Meta-analysis of the association between serum NT-pro-BNP levels and incident T2D.

We combined our result with the estimate of the FINRISK97 study, which did not exclude baseline cardiovascular disease [11], since there was no evidence for heterogeneity across the two studies (*I* ^2^ = 0%, *p* = 0.74). The difference of one SD in log-transformed NT-pro-BNP levels was associated with an 18% reduced risk of T2D (HR = 0.82 95% CI 0.74–0.90, *p* = 0.0001) in the meta-analysis comprising 857 T2D cases and 8,150 non-cases (Figure 2).

### Association of rs198389 with B-Type Natriuretic Peptide Levels and Cardio-Metabolic Traits

**Figure 3:**
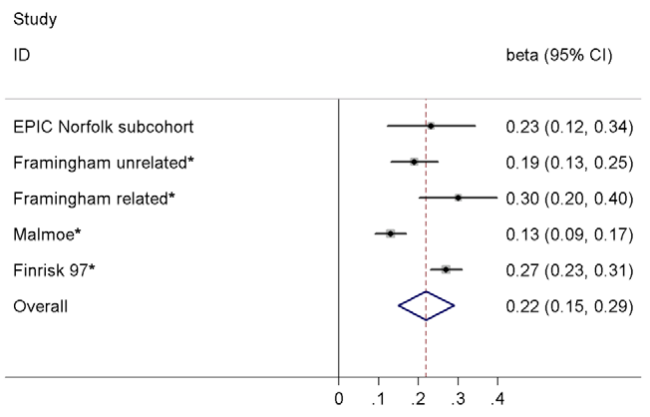
Meta-analysis of the association between the variant rs198389 and serum BNP levels. Effect estimates (beta) are from linear regression assuming an additive model and are shown on the SD scale. Asterisk indicates that proxy rs632793 was used.

In the EPIC-Norfolk subcohort excluding participants with incident T2D (final *n* with available genotype = 650), each copy of the C allele of rs198389 was associated with an increase of 0.23 SD (95% CI 0.12–0.34, *p*<0.001) in log-transformed NT-pro-BNP levels. We combined our result with a previously published meta-analysis [6], comprising in total 15,123 individuals (Figure 3). In pooled analysis we observed an increase of 0.22 SD (95% CI 0.15–0.29, *p*<0.001) in log-transformed BNP/NT-pro-BNP levels per allele, with estimates ranging from 0.13 to 0.30 SD and evidence for significant heterogeneity across studies (*I* ^2^ = 86.1%, *p*<0.001). Heterogeneity was driven by the estimate of the Malmoe study (from [6]); when excluding this study from the meta-analysis, the pooled estimate as well as the calculated expected effect of rs198389 on T2D risk did not change markedly (data not shown).

In the total EPIC-Norfolk cohort excluding participants with prevalent T2D there was no evidence for a significant association between the genotype of rs198389 and cardio-metabolic characteristics including BMI; waist circumference; systolic and diastolic blood pressure; total, low-density lipoprotein, and high-density lipoprotein cholesterol; triglycerides; alcohol consumption; levels of serum uric acid, serum creatinine, and C-reactive protein; history of hypertension; smoking; and family history of diabetes (*n* between 15,049 and 19,746), although there was a borderline significant association of lower systolic and diastolic blood pressure and a lower rate of history of hypertension with the C allele of rs198389 (all *p* = 0.07; Table S2).

### Association between rs198389 and Type 2 Diabetes Risk

**Figure 4:**
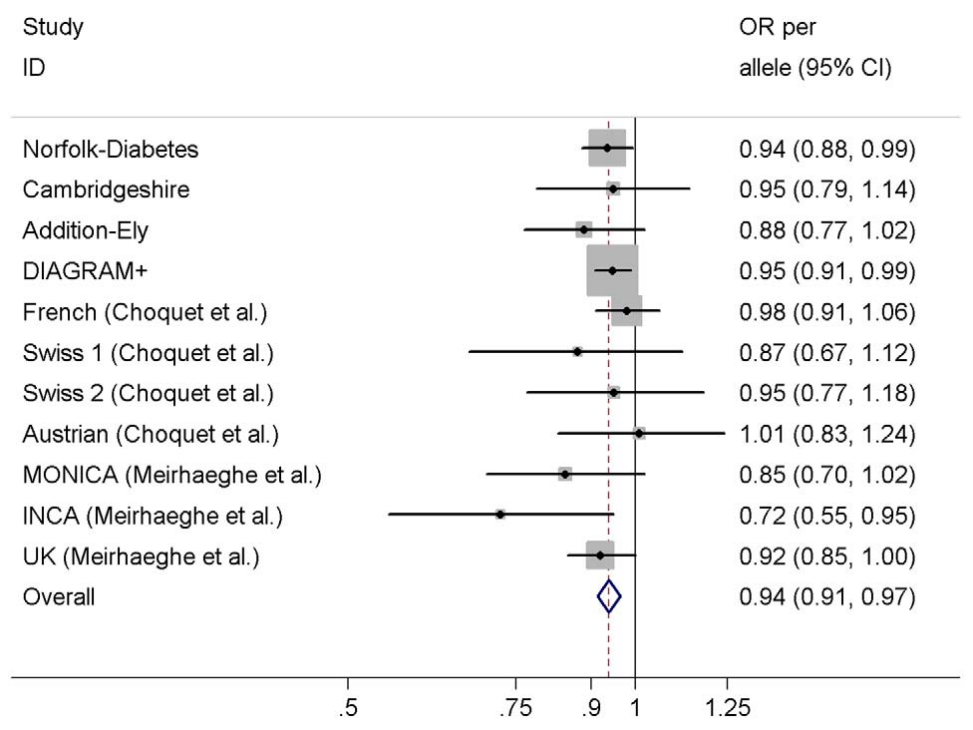
Meta-analysis of the association between the variant rs198389 and risk of T2D. Adjusted for age, sex, and BMI except for MONICA (additionally adjusted for three centres) and UK (unadjusted).

We observed a significant association between the variant rs198389 and risk of T2D in a meta-analysis of our three case-control studies, DIAGRAM+, and seven previously published case-control studies comprising a total of 23,382 T2D cases and 57,898 controls (Figure 4), with an OR of 0.94 (95% CI 0.91–0.97, *p*<0.001) per each C allele of rs198389. There was no evidence for heterogeneity across studies (*I* ^2^ = 0%, *p* = 0.64).

We used the estimates of the association between (a) the variant rs198389 and NT-pro-BNP levels and (b) NT-pro-BNP levels and risk of T2D to calculate an approximate expected effect of the variant rs198389 on T2D (Figure 1). The expected effect size was 0.96 (95% CI 0.93–0.98) per C allele of rs198389, which was similar to that observed in the case-control studies.

To estimate the unconfounded association of NT-pro-BNP levels with T2D risk we performed an instrumental variable analysis in the EPIC-Norfolk case-cohort data. We observed an OR for T2D of 0.89 (95% CI 0.37–2.16) per one SD increase in log-transformed NT-pro-BNP levels, which was not statistically significant (*p* = 0.80) because of the small sample size available for this analysis. However, it is consistent in size and direction with the estimate of the main observational association analysis (HR = 0.82, 95% CI 0.74–0.90).

## Discussion

In a large prospective cohort free of baseline T2D and cardiovascular disease, we demonstrate an inverse association between NT-pro-BNP levels and risk of incident T2D independently of several established risk factors. In a Mendelian randomization approach we integrated meta-analysed risk estimates of the triangulation between genetic variant rs198389, NT-pro-BNP levels, and T2D risk and provide evidence for a potential causal, protective role of the BNP hormone system in the aetiology of T2D. The association between the variant rs198389 and risk of T2D expected from the NT-pro-BNP to T2D association and the difference in NT-pro-BNP levels per rs198389 allele was similar to that observed in T2D case-control studies, and instrumental variable analysis suggested an effect size of genetically increased NT-pro-BNP levels on risk for T2D consistent with that found in the ordinary regression analysis of observational cohort studies.

Our results for a prospective cohort on the inverse association between NT-pro-BNP levels and T2D risk are in line with previous cross-sectional data [3] and a very recently published analysis of the FINRISK97 study in which the prospective association of 31 biomarkers, including BNP and NT-pro-BNP, with T2D was examined [11]. The association of NT-pro-BNP with T2D risk was stronger in our study compared to in FINRISK97, but we did not detect significant heterogeneity in a meta-analysis of both studies. The stronger association seen in our study might be due to our exclusion of prevalent cardiovascular disease, which leads to a release of NT-pro-BNP into circulation and hence might dilute the association of NT-pro-BNP levels with T2D in FINRISK97. We did not assess cardiac function in our study and used self- report to exclude prevalent cardiovascular disease. Given that latent left-ventricular dysfunction is common in elderly populations [26], we still may have underestimated the association of NT-pro-BNP with T2D, and in consequence, expected and observed associations between rs198389 and T2D might indeed be identical.

Although we used a prospective cohort with a long follow-up time and multivariable adjustment, bias by reverse causality or residual confounding cannot be completely ruled out. For instance, disease processes such as insulin resistance might precede the diagnosis of T2D for many years [27] and may also affect NT-pro-BNP levels. However, the novelty of our study is the integration of new and existing data in a Mendelian randomization approach, which allows a more definite conclusion on the likelihood of the causal nature of associations observed, similar to randomized controlled trials, because randomly allocated genetic variants are not expected to be subject to confounding or reverse effects [23]. We used a genetic variant within the BNP gene locus (rs198389) for which a significant association with risk of T2D was previously reported [5],[19]. These earlier studies proposed a recessive model for the effect of rs198389 on T2D risk, based on the initially observed association with fasting glucose levels and levels of statistical significance for the association with T2D. We used an additive model, though, which is unequivocally supported by observed associations with hormone levels, and thus might better reflect underlying physiology, assuming a linear relation between hormone levels and effects. Exceeding the sample size of the previous meta-analysis by almost 32,000, individuals including 11,000 T2D cases, we have good statistical power to reliably estimate the risk of T2D associated with the rs198389 genotype. Accordingly, our estimation of the genotype to NT-pro-BNP level association is also based on more than 15,000 individuals.

An important assumption of Mendelian randomization is that the genetic variant must mediate its effect on outcome only via the risk factor, i.e., the genetic variant shows no pleiotropic effects. Although this assumption cannot be proven formally in practice because of incomplete knowledge of the underlying biology, we did not observe significant associations between the variant rs198389 and potential confounders in an analysis of about 20,000 individuals. Notably, rs632793, which was used as a proxy for rs198389 in some of our analyses, is not only associated with NT-pro-BNP levels but also with atrial natriuretic peptide (ANP) levels. Stimuli for hormone secretion are similar for ANP and BNP, and both hormones share the same receptors for mediating physiological effects. ANP and BNP hormone levels are highly correlated (*r* = 0.71), and coordinate regulation at the genetic level has been proposed [6],[11]. This strong correlation makes it difficult to disentangle the distinct effects of ANP and BNP. The possible association between rs198389 and blood pressure might be mediated through ANP, which was shown to be robustly associated with blood pressure in humans [6], but there might also be a weak effect of BNP on blood pressure. However, a potential association with blood pressure would not affect our main conclusions, as it is unlikely that hypertension is on the causal pathway for development of T2D.

Additionally, there is evidence in support of BNP mediating the observed association between rs198389 and T2D rather than ANP. First, the proxy rs632793 is associated with ANP levels, but the association with BNP levels is almost three times stronger [6]. Second, the variant rs198389 is within the promoter region of the BNP gene and has been shown to influence promoter activity in experimental studies [5], which suggests that rs198389 is functionally relevant for regulating BNP hormone levels. Third, preliminary analysis on a genetic variant (rs5068) within the ANP locus that has an effect on BNP levels similar to that of rs198389 and an almost 3-fold stronger effect on ANP levels also showed an effect on T2D risk similar to that of rs198389 (data not shown). Given that the effects of ANP and BNP levels on T2D risk are similar [11], the latter suggests that the association between rs5068 and T2D is also mediated through BNP rather than through ANP levels. However, because of the limited specificity of our instrumental variable, we cannot rule out a role of ANP in the development of T2D. Analysis in other ethnic groups with a different linkage disequilibrium structure between ANP and BNP genotypes might help clarify the distinct role of both hormone systems in T2D.

There are additional limitations to this study. Our cohorts comprised only individuals of European descent, which limits generalisability of our findings to other ethnicities. Furthermore, we cannot provide conclusive evidence for the underlying mechanism of the association between the BNP hormone system and T2D. Further experimental study might help point to potential underlying mechanisms, which then can be more specifically tested in genetic epidemiological studies. Finally, the statistical power of our instrumental variable analysis within the EPIC-Norfolk cohort was not sufficient to conclude or refute a potential causal association between the BNP hormone system and T2D on its own. However, effect estimates of the instrumental variable analysis and the ordinary regression analysis were consistent, providing evidence for the validity of observational results and, hence, for a potential causal association.

Our findings provide insight into the pathophysiology of T2D by suggesting that the BNP hormone system might have a protective role, and are in line with existing experimental evidence. Transgenic mice over-expressing BNP and components of the BNP downstream signalling cascade were protected from diet-induced insulin resistance and obesity compared to wild-type mice, by up-regulation of mitochondrial biogenesis and fat oxidation [28]. Furthermore, natriuretic peptide receptors are shown to be expressed in pancreatic beta-cells [29]. An in vitro study in mice showed that activation of the natriuretic peptide receptor-A directly modulates pancreatic beta-cell function by blocking ATP-dependent potassium channel activity, increasing glucose-elicited Ca^2+^ signals, and enhancing glucose-stimulated insulin secretion in islets of Langerhans [30].

Our findings might have implications for future study by directing research on exploration of the physiological role of the BNP and also the ANP hormone system. So far, beyond the cardiovascular and lipolytic effects, little is known about why a cardiovascular hormone such as BNP would be physiologically linked to metabolism. It is well known that BNP is released in response to physical exercise [31], and thus might contribute to satisfy the increased energy demand via its lipolytic activity. However, the physiological background for the link to glucometabolic regulation remains to be determined. Furthermore, the evidence for a potential causal link between the BNP hormone system and T2D also promotes BNP as a potentially interesting target of preventive interventions. Influencing BNP activity by pharmaceutical interventions has been proven to be feasible in the context of cardiovascular medicine, e.g., by using recombinant BNP (Nesiritide) or modifying BNP cleavage and signalling [32],[33].

In conclusion, using a Mendelian randomization approach our study provides evidence for a potential beneficial role of the BNP hormone system in the aetiology of T2D. Further studies are needed to explore underlying mechanisms.

## Supporting Information

Table S1 **Baseline characteristics of the EPIC-Norfolk T2D case-cohort, by case status.**
(DOC)

Table S2 **Baseline characteristics of non-diabetic participants of the EPIC-Norfolk cohort, by rs198389 genotype.**
(DOC)

## Abbreviations

ANP: atrial natriuretic peptide
BMI: body mass index
BNP: B-type natriuretic peptide
DIAGRAM+: Diabetes Genetics Replication and Meta-Analysis +
HR: hazard ratio
MRC: Medical Research Council
NT-pro-BNP: N-terminal fragment of pro-BNP
OR: odds ratio
SD: standard deviation
T2D: type 2 diabetes
