# Supplementary material for: Mendelian Randomization Study of B-Type Natriuretic Peptide and Type 2 Diabetes: Evidence of Causal Association from Population Studies
Source: PLoS Med. 2011 Oct 25;8(10):e1001112. doi: 10.1371/journal.pmed.1001112 (PMC3201934; doi:10.1371/journal.pmed.1001112)
Supplement: Table S1 — Baseline characteristics of the EPIC-Norfolk T2D case-cohort, by case status. (DOC) [file pmed.1001112.s001.doc]

|  | Controls  (n=740) | Cases  (n=440) | p-value* |
| --- | --- | --- | --- |
| Age, year | 57.7 (9.3) | 60.9 (8.4) | <0.0001 |
| Sex, male | 286 (38.7%) | 227 (51.6%) | <0.0001 |
| BMI, kg/m2 | 25.9 (3.6) | 29.3 (4.4) | <0.0001 |
| Waist circumference, cm | 86.0 (11.7) | 97.1 (12.4) | <0.0001 |
| Systolic blood pressure, mmHg | 134 (18) | 143 (18) | <0.0001 |
| Diastolic blood pressure, mmHg | 81.6 (11.2) | 86.4 (11.4) | <0.0001 |
| Cholesterol, mmol/l | 6.12 (1.12) | 6.20 (1.19) | 0.27 |
| LDL cholesterol, mmol/l | 3.94 (1.01) | 3.97 (1.07) | 0.61 |
| HDL cholesterol, mmol/l | 1.45 (0.42) | 1.21 (0.34) | <0.0001 |
| Triglyceride, mmol/l | 1.62 (0.84) | 2.24 (1.02) | <0.0001 |
| Serum uric acid, µmol/l | 298 (75) | 339 (88) | <0.0001 |
| Hx hypertension | 79 (10.7%) | 102 (23.2%) | <0.0001 |
| Family history of diabetes | 79 (10.7%) | 113 (25.7%) | <0.0001 |
| Smoking | 104 (14.1%) | 44 (10.0%) | 0.04 |

Results are mean (SD), or percentage.

* p=value is calculated by ANOVA for continuous variables and Chi^2^-test for binary variables
