# Supplementary material for: Mendelian Randomization Study of B-Type Natriuretic Peptide and Type 2 Diabetes: Evidence of Causal Association from Population Studies
Source: PLoS Med. 2011 Oct 25;8(10):e1001112. doi: 10.1371/journal.pmed.1001112 (PMC3201934; doi:10.1371/journal.pmed.1001112)
Supplement: Table S2 — Baseline characteristics of non-diabetic participants of the EPIC-Norfolk cohort, by rs198389 genotype. (DOC) [file pmed.1001112.s002.doc]

|  | rs189389 genotype | | | |  |
| --- | --- | --- | --- | --- | --- |
|  | N  (max. 19,746) | TT  (n=6,528) | TC  (9,647) | CC  (3,571) | p-value* |
| Age, year | 19,746 | 58.5 (9.2) | 58.7 (9.3) | 59.1 (9.4) | 0.01 |
| Sex, male | 19,746 | 48.0 | 49.7 | 48.3 | 0.50 |
| BMI, kg/m2 | 19,718 | 26.3 (3.7) | 26.2 (3.7) | 26.3 (3.8) | 0.99 |
| Waist circumference, cm | 19,729 | 88 (12) | 89 (12) | 89 (12) | 0.92 |
| Systolic blood pressure, mmHg | 19,708 | 136 (18) | 135 (18) | 135 (18) | 0.07 |
| Diastolic blood pressure, mmHg | 19,708 | 82.7 (11.2) | 82.6 (11.2) | 82.4 (11.2) | 0.07 |
| Cholesterol, mmol/l | 19,157 | 6.18 (1.15) | 6.18 (1.18) | 6.17 (1.16) | 0.36 |
| LDL cholesterol, mmol/l | 18,524 | 3.97 (1.03) | 3.97 (1.04) | 3.96 (1.03) | 0.26 |
| HDL cholesterol, mmol/l | 18,523 | 1.41 (0.45) | 1.41 (0.41) | 1.40 (0.42) | 0.70 |
| Triglyceride, mmol/l | 19,155 | 1.82 (1.10) | 1.81 (1.08) | 1.84 (1.10) | 0.84 |
| Alcohol consumption, units/week | 19,746 | 7.3 (9.5) | 7.3 (9.8) | 7.4 (9.6) | 0.60 |
| Serum uric acid, µmol/l | 15,123 | 296 (82) | 298 (83) | 297 (82) | 0.96 |
| Serum creatinine, µmol/l | 15,049 | 87 (21) | 87 (24) | 87 (20) | 0.99 |
| C-reactive protein, mg/l | 15,133 | 3.0 (5.6) | 3.1 (6.9) | 3.2 (7.0) | 0.96 |
| Hx myocardial infarction | 19,732 | 2.9 | 3.0 | 3.1 | 0.98 |
| Hx cerebrovascular disease | 19,734 | 1.2 | 1.3 | 1.4 | 0.52 |
| Hx hypertension | 17,718 | 13.7 | 14.1 | 12.6 | 0.07 |
| Family history of diabetes | 19,726 | 12.1 | 12.3 | 13.1 | 0.12 |
| Smoking | 19,579 | 11.9 | 11.6 | 12.4 | 0.46 |

Results are mean (SD), or percentage.

* p=value is calculated from age- and sex-adjusted linear regression for continuous variables, and from age- and sex-adjusted logistic regression for categorical variables, except for variables age and sex.
